# The leishmanicidal activity of oleuropein is selectively regulated through inflammation- and oxidative stress-related genes

**DOI:** 10.1186/s13071-016-1701-4

**Published:** 2016-08-09

**Authors:** Ioannis D. Kyriazis, Olga S. Koutsoni, Nektarios Aligiannis, Kalliopi Karampetsou, Alexios-Leandros Skaltsounis, Eleni Dotsika

**Affiliations:** 1 Laboratory of Cellular Immunology, Hellenic Pasteur Institute, 127 Vas. Sofias Ave., 11521 Athens, Greece; 2 Department of Pharmacognosy and Natural Products Chemistry, Faculty of Pharmacy, National and Kapodistrian University of Athens, Panepistimiopolis Zografou, 15771 Athens, Greece; 3 VIVUS research and diagnostic center, 160 Konstanta str, Volos, Greece

**Keywords:** Oleuropein, *Leishmania*, Visceral leishmaniasis, Immunomodulation, Oxidative stress, Inflammation

## Abstract

**Background:**

Much research effort has been focused on investigating new compounds derived from low-cost sources, such as natural products, for treating leishmaniasis. Oleuropein derived from numerous plants, particularly from the olive tree, *Olea europaea* L. (*Oleaceae*), is a biophenol with many biological activities. Our previous findings showed that oleuropein exhibits leishmanicidal effects against three *Leishmania* spp. in vitro, and minimizes the parasite burden in *L. donovani*-infected BALB/c mice. The aim of the present study is to investigate the possible mechanism(s) that mediate this leishmanicidal activity.

**Methods:**

We determined the efficacy of oleuropein in elevating ROS and NO production in *L. donovani*-infected J774A.1 macrophages and in explanted splenocytes and hepatocytes obtained from *L. donovani*-infected BALB/c mice. We also assessed the expression of genes that are related to inflammation, T-cell polarization and antioxidant defense, in splenocytes. Finally, we determined the ratios of specific IgG2a/IgG1 antibodies and DTH reactions in *L. donovani-*infected BALB/c mice treated with oleuropein.

**Results:**

Oleuropein was able to elevate ROS production in both in vitro and in vivo models of visceral leishmaniasis and raised NO production in *ex vivo* cultures of splenocytes and hepatocytes. The extensive oxidative stress found in oleuropein-treated mice was obviated by the upregulation of the host’s antioxidant enzyme (m*GCLC*) and the simultaneous downregulation of the corresponding enzyme of the parasite (Ld*GCLC*). Moreover, oleuropein was able to mount a significant Th1 polarization characterized by the expression of immune genes (*IL-12β*, *IL-10*, *TGF-β1*, *IFN-γ*) and transcription factors (*Tbx21* and *GATA3*). Moreover, this immunomodulatory effect was also correlated with an inhibitory effect on *IL-1β* gene expression, rather than with the expression of *IL-1α*, *IL-1rn* and *TNF-α*. Furthermore, oleuropein-treated BALB/c mice mounted a delayed-type hypersensitivity (DTH) response and an elevated *Leishmania*-specific IgG2a/IgG1 ratio that clearly demonstrated an in vivo protective mechanism.

**Conclusion:**

The ability of Oleuropein to promote a Th1 type immune response in *L. donovani*-infected BALB/c mice points towards the candidacy of this bioactive compound as an immunomodulatory agent that may complement therapeutic approaches to leishmaniasis.

## Background

Leishmaniasis is a parasitic disease with diverse clinical manifestations. The parasites of the genus *Leishmania* have developed the ability to manipulate the cells of the host immune system and to survive within the macrophage phagolysosome*.* After establishment, *Leishmania* infection *per se* is able to alter the immunological and inflammatory host responses to its own benefit [1, 2]. To date, much research effort has been directed towards the discovery of new therapeutic agents capable of killing the majority of *Leishmania* species and promoting the host immune response*.* This activity of potent therapeutic agents must rely on the generation of a strong immune response orchestrated by both innate and adaptive immunity against *Leishmania* infection. The differentiation and proliferation of specific CD4^+^ T cells (T-helper cells) into different effector cell subpopulations have been recognized. Indicatively, Th1, Th2, and Th3 have been identified in murine visceral leishmaniasis through their landmark produced cytokines, interleukin-12 (IL-12), IL-10, and transforming growth factor-β (TGF-β), respectively. There has been a consensus that a Th1 dominant response over that of Th2, is responsible for the activation of macrophages that eliminate *Leishmania* parasites via microbicidal molecules, such as reactive oxygen species (ROS) and nitric oxide (NO) [3]. The polarization of Th cells into Th1 and Th2 effector cells is controlled by the regulation and production of the transcription factors T-box transcription factor (*Tbx21*) and trans-acting T-cell-specific transcription factor (*GATA3*), respectively [4]. The regulation of *GATA3* expression is considered essentially significant, because its downregulation allows the production of Tbx21 which is mainly stimulated by Th1-related cytokines, like IL-12 and interferon-γ (IFN-γ) [5, 6]. Moreover, *GATA-3* regulation is maintained by the presence of the immunosuppressing cytokine IL-10 and not IL-4 [7, 8]. On the contrary, the presence of TGF-β can halt the differentiation and the proliferation of immature T-cells into the above discrete subpopulations. These differential immune responses are also correlated with the existence of inflammatory messengers after the onset of disease or during parasite dissemination that will render a strengthened Th2-Th3 immune response allowing the gradual spread of the disease [9–12].

The Th1 immune response induces macrophages to generate leishmanicidal molecules, such as ROS and reactive nitrogen intermediates (RNI), like NO [13]. Among the various types of ROS, superoxide anion (O_2_^-^) is largely produced at the establishment of infection during the penetration of promastigotes to macrophages, while lower amounts are produced during the outspread of disease and the infection of adjacent monocytes with amastigotes. It has been shown that this reduction is mainly due to a NADPH oxidase deficiency that is being imposed by the intracellular parasite [14–16]. NO is the other anti-leishmanial molecule and its production is catalyzed by the inducible nitric oxide synthase enzyme (iNOS) from L-arginine. Contrary to ROS, NO is produced in the macrophage response against the parasites already present within the cell [17]. This is due to the fact that iNOS induction and its transformation into an active form requires at least 6 h after synergism of various stimuli, such as cytokines (IL-12, IL-18, IFN-γ, tumor necrosis factor-α; TNF-α), microbial products and elements such as lipopolysaccharide (LPS), co-stimulatory molecules, adhesion molecules, as well as immune complexes [6, 18]. *Leishmania* spp. parasites possess the glutamate-cysteine ligase enzyme (GCL or γ-GCS), which is involved in biosynthesis of the antioxidant molecule named trypanothione (TSH). TSH confers control on the oxidative potential within the host’s phagolysosomes, which allows the parasites to avoid the deleterious effects of ROS and NO [19]. On the other hand, host cells have similar molecules like glutathione (GSH) and an analogue “host” GCL [20] which protect them from extensive oxidative stress that occurs during the defense against phagocytized parasites and the production of microbicidal molecules [16, 21]. Protozoans of the genus *Leishmania* prefer TSH for their protection against ROS and NO because TSH has a 600-fold higher affinity binding to NO than GSH [22, 23]. Nevertheless, transgenic promastigotes that were heterozygous for GCL produced reduced levels of TSH and became vulnerable to oxidative stress in vitro and exhibited reduced survival within activated macrophages [23].

The activation of macrophages and the subsequent production of ROS and NO are inextricably associated with the host’s defense against leishmaniasis. However, this production must be accompanied by an acute regulation of antioxidant enzymes to defend against the oxidative burst. The differential regulation of the host and parasite antioxidant systems results in an armored protection for host cells, and in contrast, an impaired defense for the parasites. Throughout the literature, several natural products have been tested for their ability to increase the production of ROS and/or NO in in vitro and in vivo experimental models of leishmaniasis. Some natural plant products, including taxoid 10-deacetylbacattin-III [24], monoterpene linalool [25], 2′,6′-dihydroxy-4′-methoxychalcone [26], and crude leaf extracts from *Chenopodium ambrosioides* L. (*Amaranthaceae*) [27], were shown to stimulate macrophages to increase NO production during *Leishmania* infection. Furthermore, some natural animal products, like the venom from *Bungarus caeruleus* Schneider, 1801 (Elapidae) were shown to promote ROS and NO production in an in vivo experimental visceral leishmaniasis model [28].

Oleuropein (Ole), a secoiridoid present in olives and leaves of *Olea europaea* L. (*Oleaceae*), was previously shown to induce reduced parasite burden in BALB/c mice infected with *Leishmania donovani* (Laveran & Mesnil, 1903) (Trypanosomatidae) even six weeks after the termination of treatment [29]. Moreover, it has been shown that Ole is able to induce apoptotic mechanisms in several cancer cell lines without regulating the expression of molecules involved in the NF-kB signaling pathway (e.g. MAPK cascade proteins, IkB-α) [30, 31]. Furthermore, Ole has been shown to be able to refill the cellular antioxidant pool by upregulating glutathione-recovery enzyme expression [32] and to decrease inflammatory mediator production (IL-1β) by human whole blood cultures [33]. The anti-inflammatory efficacy of Ole might contribute to *Leishmania* spp. clearance, because IL-1β abrogation results in Th1 polarization [34].

In the present study, we aimed to determine the possible mechanism by which Ole is able to promote antileishmanial activity in J774A.1 macrophages infected by *L. donovani* and in target tissues (spleen and liver) of *L. donovani*-infected BALB/c mice. This study elucidates the selective anti-inflammatory ability of Ole, which favours a Th1 type cell polarization of the susceptible mouse strain challenged with viscerotropic *Leishmania* parasites. This Th1 polarized immune response is illustrated by the regulation of Th1 specific genes such as *IL-12β*, *IFN-γ* and *TNF-α* over Th2- and Th3-related genes such as *IL-10* and *TGF-β1* [35, 36]. The Th1 dominance is further confirmed by the elevated ratios of *Tbx21*/*GATA3* transcription factors and *Leishmania*-specific IgG2a/IgG1 antibodies in the spleen and serum of infected BALB/c mice treated with Ole. The above cascade induces the in vivo production of microbicidal molecules like ROS and NO. Moreover, in this study we demonstrated the ability of Ole to promote host antioxidant defense mechanism(s) in the host, whereas it is able to downregulate the corresponding parasite antioxidant defense. These findings were further complemented with the detection of delayed-type hypersensitivity response (DTH) in Ole-treated and *L. donovani*-infected BALB/c mice.

## Methods

### Parasite culture

The viscerotropic *Leishmania* strain *L. donovani* (zymodeme MON-2, strain MHOM/IN/1996/THAK35) was kindly provided by Dr. K. Soteriadou (Laboratory of Molecular Parasitology, Hellenic Pasteur Institute, Greece). Promastigotes were cultured in complete RPMI-1640 medium which consisted of RPMI-1640 with low phenol red content (Biochrom AG, Berlin, Germany), supplemented with 2 mM L-glutamine, 10 mM HEPES, 24 mM NaHCO_3_, 100 U/ml penicillin, 100 μg/ml streptomycin, and 10 % v/v heat-inactivated fetal bovine serum (FBS; Gibco, Paisley, UK). Promastigotes were grown at 26 °C in a cell culture flask.

### Macrophage culture and in vitro infection protocol

The immortalized macrophage cell line J774A.1 was purchased from the American Type Culture Collection (ATCC; Rockville, MD, USA/ ATCC No: TIB-67). The J774A.1 cells were cultured in culture flasks with complete RPMI-1640 medium and were incubated at 37 °C in a 5 % CO_2_ environment. The viability of J774A.1 cells was determined by Trypan blue staining and cells were counted in a Malassez hemocytometer. Adherent J774A.1 cells were infected in vitro with *L. donovani* stationary-phase promastigotes at a ratio of 1:15 for 4 h, as previously described [29].

### Natural product

Oleuropein (Ole) with purity of above 95 %, was extracted from air-dried, pulverized leaves (5 kg) of *Olea europaea* var *koroneiki* collected in Crete (Greece) [29]. Ole was diluted in distilled water, Millipore filtered with a 0.45 μm pore size filter (Millipore, Massachusetts, USA) and stored at 4 °C.

For the conduct of the in vitro experiments, Ole was used at two different concentrations, 128.4 μM (69.4 μg/ml), which represents the half maximal inhibitory concentration (IC_50_) against *L. donovani* promastigotes of logarithmic phase and 256.8 μM (138.8 μg/ml; i.e. 2× IC_50_) [29].

### Production of soluble *Leishmania* antigen

Soluble *Leishmania* Antigen (SLA) was derived from 10^9^ *L. donovani* stationary phase promastigotes. Briefly, promastigotes were placed in sterile phosphate buffered saline (PBS) and then disrupted with three repeated freeze-thaw cycles (freezing at -80 °C and thawing at 37 °C). Lysed parasites were then sonicated 3 times for 1 min, at 30-s intervals. The crude lysate was centrifuged at 8,000×* g* for 30 min at +4 °C, and the supernatant was aliquoted and stored at -80 °C until use. The protein concentration of SLA was measured with a NanoDrop 2000 spectrophotometer (Thermo Fischer Scientific, Waltham, MA).

### In vivo visceral leishmaniasis experimental protocol

Age-matched 8 to 10-week-old female BALB/c mice (20–25 g) were obtained from the breeding unit of the Hellenic Pasteur Institute (HPI, Athens, Greece). Experimental protocols were approved by the Animal Bioethics Committee of the HPI, according to the regulations of the European Commission Directive 1986/609 and the National Law of 1992/2015.

Briefly, BALB/c mice were infected intravenously in the lateral vein with 1.5 × 10^7^ *L. donovani* stationary-phase promastigotes. At 15 days post-infection, animals were randomly assigned to five groups. In the first three experimental groups (G1, G2, G3), Ole was administered intraperitoneally at three different concentrations (45, 15, or 5 mg/kg body weight (b.w.) of pure Ole, respectively), every other day, for up to 28 days. The fourth experimental group (G4), representing the positive control group, received miltefosine (HePC), the only drug approved by the Food and Drug Administration agency of USA (USFDA) for treating leishmaniasis. HePC (4 mg/kg b.w.), was administered by daily oral gavage for up to 28 days. BALB/c mice of the fifth group (G5 infected control group) did not receive any treatment. Finally, a group of non-infected mice (G6) served as the healthy control group (negative control group). Spleens, livers, and blood serum samples were obtained at 3 days and 6 weeks after treatment termination.

### Assessment of generalized intracellular oxidative stress

To detect general intracellular oxidative stress, 10^7^ cells/ml of each in vitro or in vivo experimental group were incubated with 5 μM of a ROS-sensitive fluorescent probe (CM-H_2_DCFDA; a chloromethyl derivative of 2′, 7′-dichlorodihydrofluorescein diacetate, Invitrogen - Molecular Probes™) for 45 min in the dark, at 37 °C in a 5 % CO_2_ environment.

More specifically, for the in vitro assays, J774A.1 cells (*L. donovani*-infected and non-infected) were incubated for 24 h with Ole (69.4 or 138.8 μg/ml) either alone or in the presence of LPS (1 μg/ml); LPS (1 μg/ml) alone; or LPS (1 μg/ml) plus IFN-γ (1 ng/ml) which represent the positive control groups of the applied method.

Concerning the in vivo assays, explanted splenocytes were directly incubated with CM-H_2_DCFDA, except for splenocytes derived from the mice of the negative-control group, which were incubated for 5 min with hydrogen superoxide (10 μM, H_2_O_2_), before incubation with CM-H_2_DCFDA. The cells of each experimental group were washed with PBS and then placed at 4 °C.

Subsequently, 10,000 cells per experimental group were analyzed with Fluorescence-Activated Cell Sorting (FACS; on a FACS Calibur apparatus, Becton-Dickinson, San Jose, CA, USA). ROS activity was evaluated in terms of the geometric mean fluorescence intensity (gMFI). The results were plotted with Cell Quest (Becton Dickinson) and Flowjo (TreeStar Inc., Ashland, USA) software.

### Quantification of extracellular nitric oxide (NO)

NO levels in the supernatants of all experimental groups were determined with the Griess reaction (Sigma-Aldrich, USA), as described before [37].

J774A.1 cells were incubated for 24 h with 69.4 or 138.8 μg/ml of Ole, either alone or in combination with LPS (1 μg/ml), LPS (1 μg/ml) alone, or LPS (1 μg/ml) plus IFN-γ (1 ng/ml). The stimuli described above were placed before and after the *L. donovani* infection for up to 24 h and NO levels were determined in the supernatant of the cultures. NO levels were also determined in uninfected J774A.1 cells treated with the same stimuli.

*Ex vivo* splenocytes and hepatocytes were cultured in the presence of complete RPMI-1640 medium and NO levels were determined after 24 h. Furthermore, splenocytes and hepatocytes obtained from the non-infected mice (G6 group) were incubated in the presence of LPS (1 μg/ml) or LPS (1 μg/ml) plus IFN-γ (1 ng/ml).

The optical density of the Griess reaction products was measured at 570 nm with a spectrophotometer (MRX, DYNATECH Laboratories, Guernsey, England).

### Gene expression analysis in splenocytes of *L. donovani-*infected BALB/c mice

Splenocytes were obtained from *L. donovani*-infected BALB/c mice of all experimental groups, 3 days after treatment termination. Total mRNA was isolated with an RNeasy Mini kit (Qiagen, Germany), and mRNA concentrations were measured with a NanoDrop 2000 spectrophotometer (Thermo Fischer Scientific). Messenger RNA was then reverse-transcribed using a SuperScript II kit (Invitrogen - Molecular Probes™) and oligo-dTs (Promega, WI, USA), and all reactions included the recombinant ribonuclease inhibitor, RNaseOUT™ (Invitrogen).

cDNAs of all the in vivo experimental groups were evaluated with real time PCR, performed using an Exicycler 96 thermocycler (Bioneer, Daejeon, Korea), using the Kapa SYBR Fast Universal 2× qPCR Master Mix kit (Kapa Biosystems Ltd., London, UK). The specific primers for the interleukin-1α (*IL-1α*), interleukin-1β (*IL-1β*), interleukin-1 receptor antagonist (*IL-1rn*), interleukin-10 (*IL-10*), subunit 2 of interleukin-12 (*IL-12β*), interferon-γ (*IFN-*γ), tumor necrosis factor-α (*TNF-α*), transforming growth factor beta-1 (*TGF-β1*), T-box transcription factor (*Tbx21*), trans-acting T-cell-specific transcription factor (*GATA3*), subunit 2 of nuclear factor kappa-B (*NF-kB2)*, and the glyceraldehyde dehydrogenase of the 3-phosphatase (*GAPDH*) gene sequences were designed by Qiagen (QuantiTect Primer Assays; Qiagen, Venlo, Netherlands). The primers used to target the murine and *L. donovani* glutamate-cysteine ligase catalytic subunit sequences (*mGCLC* and *LdGCLC*, respectively) and the *L. donovani* a-tubulin gene sequence (*LdAtub*) were described previously [38, 39]. The PCR was conducted according to Qiagen’s PCR protocol for the QuantiTect Primer Assays. All gene expression ratios were computed with the *ΔΔCt* method [40]. All qPCR experiments were performed in 3 replicates for each experimental condition.

### Detection of *Leishmania*-specific IgG antibodies

Blood from *L. donovani*-infected BALB/c mice was collected at 3 days and 6 weeks after the termination of the treatments. Serum was separated upon centrifugation of blood at 4000× *g* for 5 min. The *Leishmania*-specific IgG1 and IgG2a antibodies were determined with an indirect ELISA method. Briefly, 96-well microtiter plates were coated with 5 μg/ml of SLA in carbonate buffer (15 mM Na_2_CO_3_, 35 mM NaHCO_3_), pH 9.6 and left overnight at 4 °C. For the detection of IgG1 and IgG2a isotypes, serum samples (1/100 dilution) were added, as described previously [41].

### Measurement of Delayed Type Hypersensitivity (DTH)

In order to determine the delayed hypersensitivity reactions, BALB/c mice that had been treated with Ole (15 mg/kg b.w.), were intradermally administered with total soluble *L. donovani* antigen (20 μg dissolved in 20 μl of sterile PBS) in the left hind footpad, 3 days post-treatment termination. As a negative control, the right hind footpad was given an equal volume of sterile PBS. The DTH response was determined by measuring the increase in thickness between the two hind footpads, for up to 48 h. Footpad swelling was measured with a dial gauge caliper (Mitutoyo, Kanagawa, Japan) and the difference was based on the following formula: Footpad swelling (mm) = thickness of left footpad – thickness of right footpad. The DTH was also determined in non-infected and in infected and non-treated BALB/c mice.

### Statistical analysis

Three independent experiments were performed in duplicate for each in vitro method used. The in vivo experimental protocol was conducted twice and each group consisted of 6 mice. Statistical differences between the means of the in vitro (*P* ≤ 0.05) and *ex vivo* (*P* ≤ 0.1) experiments were analyzed for significance with the non-parametric Mann-Whitney test.

## Results

### Oleuropein induces intracellular ROS and nitric oxide production in J774A.1 cells

**Fig. 1:**
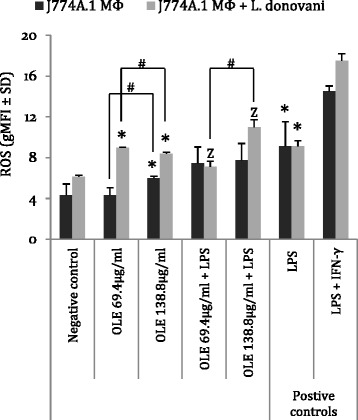
ROS levels in uninfected and *L. donovani*-infected J774A.1 macrophages. The negative control is represented by uninfected and untreated J774A.1 macrophages while the positive controls are represented by *L. donovani*-infected J774A.1 cells incubated with either LPS alone or with LPS plus IFN-γ. ^*^indicates significant differences compared to the negative control group; ^Z^indicates significant differences compared to the positive control (LPS) group; ^#^indicates significant differences between the two different concentrations of Ole

Increased intracellular levels of ROS and extracellular production of NO indicate macrophage activation via the classical pathway and parasite clearance [3]. We first determined the effect of Ole in the generation of ROS in infected or non-infected J774A.1 macrophages. Non-infected J774A.1 cells (dark grey bars in Fig. 1) that were incubated with 138.8 μg/ml of Ole exhibited significantly higher levels of intracellular ROS (gMFI = 6 ± 0.2) compared to the corresponding levels generated by untreated J774A.1 cells (gMFI = 4.3 ± 1.1, *P* = 0.05). Nevertheless, Ole was not able to strengthen the intracellular oxidative stress caused by LPS, which is a potent stimulator of macrophages. More specifically, J774A.1 cells that were incubated with Ole plus LPS, did not exhibit augmentation of intracellular ROS compared to J774A.1 cells that were stimulated only with LPS (gMFI = 9.1 ± 2.4).

Moreover, *L. donovani*-infected J774A.1 cells (light gray bars in Fig. 1) that were treated with Ole, exhibited significantly (*P* ≤ 0.05) higher intracellular ROS levels than their respective control groups. More specifically, *L.donovani*-infected J774A.1 cells that were treated with 69.4 and 138.8 μg/ml of Ole, exhibited high geometric mean fluorescence indexes (9 ± 0.1 and 8.4 ± 0.2, respectively; *P* = 0.05), when the corresponding mean for non-treated and infected J774A.1 cells was determined at 6.1 ± 0.2 gMFI (Fig. 1). Furthermore, it is noteworthy that *L. donovani*-infected J774A.1 cells that were simultaneously incubated with Ole (138.8 μg/ml) and LPS managed to exert increased ROS production (gMFI = 11 ± 0.7; *P* = 0.05) in comparison to infected cells treated only with LPS (gMFI = 9.1 ± 0.6), whereas infected cells that were treated with 69.4 μg/ml of Ole plus LPS, exhibited significantly diminished ROS production (gMFI = 7.1 ± 0.6; *P* = 0.05, Fig. 1). These data indicate the differential effect of Ole in inducing ROS production in vitro, depending on different parameters such as the concentration of Ole, the parasite challenge or the simultaneous activation with LPS.

**Fig. 2:**
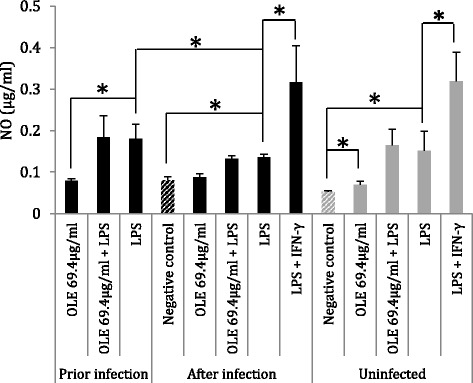
NO levels in J774A.1 macrophage culture supernatants. Infected groups represent macrophages infected with *L. donovani* promastigotes. *indicates significant differences between the two conditions indicated with brackets

In addition, in uninfected J774A.1 cells Ole promoted a significant (*P* = 0.05) increase in nitrite production (0.069 ± 0.008 μg/ml; Fig. 2) compared to the baseline level (negative control, 0.053 ± 0.002 μg/ml).

However, Ole did not augment extracellular NO levels in *L. donovani*-infected J774A.1 cells, regardless of whether it was added before or after the infection or alone or in combination with LPS (Fig. 2).

### Oleuropein induces high oxidant production in spleen and liver cells

The levels of intracellular ROS were determined in splenocytes of *L. donovani*-infected BALB/c mice, 3 days and 6 weeks after the termination of Ole or HePC treatment.

**Fig. 3:**
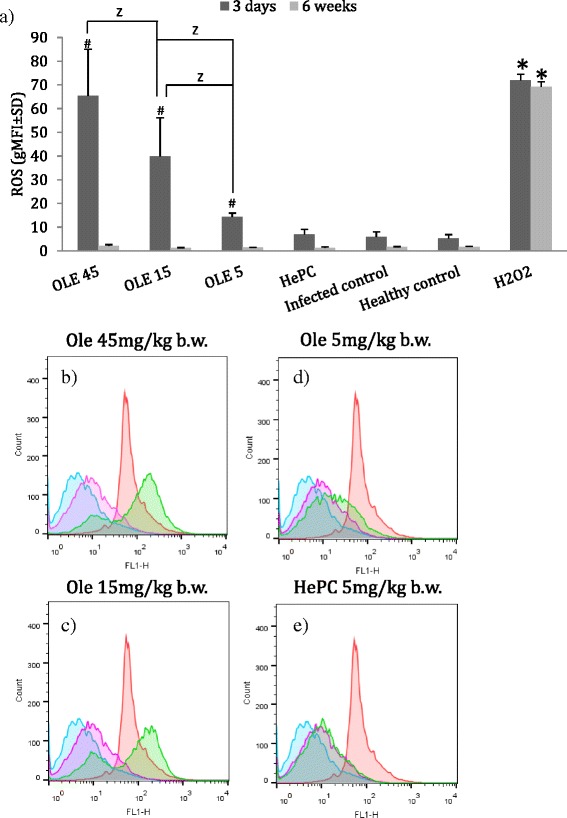
**a** Intracellular ROS levels in splenocytes of BALB/c mice at 3 days or 6 weeks after treatment termination. ^#^ and ^*^ indicate significant differences, compared to infected and healthy (negative) control groups, respectively. ^Z^ indicates statistically significant differences between the Ole-treated groups indicated with brackets. **b**-**e** Representative flow cytometry histograms derived from experimental groups at 3 days after treatment termination. *Blue* and *pink* curves represent ROS levels in healthy control and infected control groups, respectively. *Red* curves represent ROS levels induced by H_2_O_2_ in uninfected splenocytes. *Green* curves represent ROS levels in infected splenocytes that received the indicated treatments

At 3 days post-termination of treatment (dark grey bars in Fig. 3a), splenocytes exhibited a significant increase in the levels of intracellular ROS in response to Ole. Splenocytes of mice that had been treated with 45, 15 and 5 mg/kg b.w. of Ole, exhibited inflated levels of ROS reaching 65.5 ± 19.6 (*P* = 0.004), 39.8 ± 16.3 (*P* = 0.004) and 14.4 ± 1.6 gMFI (*P* = 0.004), respectively, compared to splenocytes of infected and non-treated mice (infected control group, G5; gMFI = 6.0 ± 2.0). It is noteworthy that splenocytes obtained from *L. donovani*-infected BALB/c mice treated with HePC (positive control group), did not exhibit high levels of ROS (gMFI = 7 ± 1.2; Fig. 3a). Moreover, it is important that although we observed significant production of ROS in splenocytes of Ole-treated mice at 3 days post-termination of treatment, we did not notice any significant production of ROS at 6 weeks post-termination of treatment in all experimental groups.

**Fig. 4:**
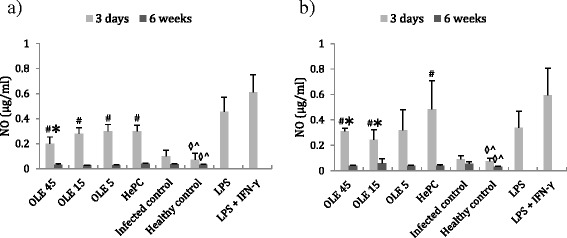
NO levels in the supernatant of splenocytes (**a**) and hepatocytes (**b**) of *L. donovani*-infected mice upon treatment with Ole or HePC. NO levels were determined at 3 days or 6 weeks after treatment termination. ^#^ and ^*^ indicate significant differences compared to the infected and positive (HePC-treated) control groups, respectively. ^◊^ and ^^^ indicate significant differences compared to explanted cells from uninfected and untreated mice that received LPS and LPS plus IFN-γ

Subsequently, we determined the levels of NO in the supernatant of spleen and liver cells that were explanted from all the in vivo experimental groups. At 3 days after treatment termination (light grey bars in Fig. 4), single cell suspensions obtained from spleen and liver tissues from Ole- or HePC-treated mice (G1-G4) showed a significant increase in NO levels compared to the infected control group (G5) (Fig. 4). As shown in Fig. 4, in splenocytes from BALB/c mice treated with Ole or HePC, the levels of NO were 0.2 ± 0.052 μg/ml for G1 (*P* = 0.004), 0.278 ± 0.048 μg/ml for G2 (*P* = 0.004), 0.299 ± 0.054 μg/ml for G3 (*P* = 0.004) and 0.299 ± 0.047 μg/ml for G4 (*P* = 0.004), whereas the corresponding levels for G5 (infected control group) and G6 (healthy control group) were 0.1 ± 0.045 and 0.07 ± 0.053 μg/ml, respectively. In addition, explanted liver cell supernatants exhibited similar nitrite production (Fig. 4b). At 3 days post-termination of treatment, only mice from G1 and G2 groups (treated with Ole 45 and 15 mg/kg b.w., respectively) showed significant increases in NO levels (0.309 ± 0.025; *P* = 0.004 and 0.241 ± 0.081 μg/ml; *P* = 0.004, respectively) compared to the infected control group (G5: 0.091 ± 0.027 μg/ml). Similarly, treatment with HePC increased significantly the levels of NO in hepatocytes (0.482 ± 0.227 μg/ml, *P* = 0.004) (Fig. 4b). Cells treated with LPS (*P* = 0.02) and LPS/IFN-γ (*P *= 0.02) produced significant amounts of NO in their supernatant compared to healthy control groups (Fig. 4a, b).

Finally, at 6 weeks post-treatment, the levels of nitrites in the supernatant of the splenocytes and hepatocytes were not significantly different among all experimental groups (Fig. 4a, b).

### Oleuropein induces *Leishmania-*specific IgG1 and IgG2a antibody production

**Fig. 5:**
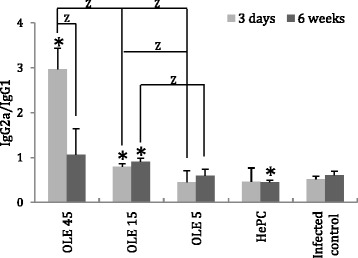
*Leishmania*-specific antibody production. The IgG2a/IgG1 ratios were determined in *L. donovani*-infected BALB/c mice upon their treatment with Ole or HePC. Ratios were measured at 3 days and 6 weeks after treatment termination. ^*^indicates significant differences compared to infected control group; ^Z^indicates significant differences between groups indicated with brackets

*Leishmania*-specifc IgG1 and IgG2a antibodies were detected in the serum of *L. donovani*-infected BALB/c mice, upon their treatment with either Ole or HePC. At 3 days post-termination of treatment, we observed a significant and dose-dependent increase of the ratio IgG2a/IgG1 in mice that were treated with Ole (light grey bars in Fig. 5). More specifically, in mice treated with 45 and 15 mg/kg b.w. of Ole (G1 and G2), the IgG2a/IgG1 ratios were 2.96 and 0.8, respectively. These ratios were 5.75-fold (*P* = 0.004) and 1.55-fold (*P* = 0.004) greater than the corresponding ratio in the infected control group (G5). These results show that B-cell antibody production in Ole-treated mice is being guided by a mixed T-cell population where Th1 phenotype is being elevated. On the other hand, HePC-treated mice did not mount a significant IgG2a increase, indicating a mixture of Th1/Th2 immune responses.

At 6 weeks after termination of treatment (dark grey bars in Fig. 5), IgG2a/IgG1 ratios in Ole-treated mice (45 and 15 mg/kg b.w.) were maintained at higher levels compared to the infected control group (G5).

### Levels of *NF-kB2,* murine *GCLC* and *L. donovani GCLC* gene expression

The presence of high oxidative stress in the tested tissues is likely to affect both the normal functioning of the host cells as well as the parasites. This toxic biochemical environment caused by the oxidative burst is accompanied by changes in transcriptional levels of antioxidant-related genes like *NF-kB2* and *GCLC*.

**Fig. 6:**
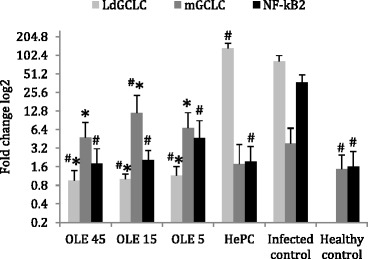
Fold changes log2 of the Ld*GCLC*, m*GCLC* and *NF-kB2* gene expression in splenocytes of *L. donovani*-infected BALB/c mice, 3 days after termination of treatments with Ole and HePC. ^#^ and ^*^ indicate significant differences compared to the infected and positive (HePC-treated) control groups, respectively

At 3 days after treatment termination, the expression levels of *NF-kB2* gene in mice of the infected control group (G5) were significantly higher (*P* = 0.004) compared to all of the other experimental groups (G1-G4 and G6; Fig. 6). Specifically, *NF-kB2* expression in infected control was 23.18-fold upregulated compared to healthy and untreated mice (G6). Ole-treated experimental groups exhibited at most 20.8-fold decrease in *NF-kB2* expression compared to the infected control group (G5).

Furthermore, as shown in Fig. 6, the m*GCLC* gene expression in mice from the infected control group (G5) tended to be higher than the corresponding expression observed in mice of the healthy control group (G6; 2.56-fold; *P* = 0.086). This is possibly attributed to the presence of uncontrolled dissemination of *L. donovani* infection. Moreover, all of the Ole-treated groups (G1, G2, G3) exhibited increased mGCLC expression compared to infected mice (G5), but only mice treated with 15 mg/kg b.w. of Ole showed a tendency for higher expression levels (3.17-fold; *P* = 0.086).

The expression of parasite *GCLC* (*LdGCLC*) was highly elevated in infected and HePC-treated mice compared to Ole-treated mice. More specifically, HePC-treated mice demonstrated a 1.62-fold upregulation (*P* = 0.014) in *LdGCLC* expression compared to the infected control. These elevated transcription levels in infected control, reflect the extreme necessity of *L. donovani* parasites to upregulate one of the most important genes involved in protection against increased oxidative stress. On the other hand, Ole exhibited a different mode of action from HePC. Although both HePC and Ole increased oxidative stress (mainly by NO production), in infected splenocytes, all Ole treatments caused a 100-fold repression (Ole45: *P* = 0.009; Ole15: *P* = 0.014; Ole5: *P* = 0.024) in *LdGCLC* expression compared to the infected control.

### Levels of *IL-1rn, IL-1β,* and *IL-1a* gene expression

Visceral leishmaniasis is a systemic inflammatory disease and its severity is determined by the capacity of the host immune system to control inflammation. The transcription of inflammation-related genes is critical for the development of such cellular micro-environment that allows or prevents parasitic spread [2]. Moreover, one well-known characteristic of Ole is its anti-inflammatory efficacy [42].

**Fig. 7:**
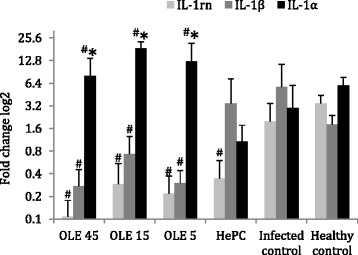
Fold changes log2 of the *IL-1rn*, *IL-1b* and *IL-1a* gene expression in splenocytes of *L. donovani*-infected BALB/c mice, 3 days after termination of treatments with Ole and HePC. ^#^ and ^*^ indicate significant differences compared to the infected and positive (HePC-treated) control groups, respectively

In this study, we demonstrated that the intraperitoneal administration of Ole in *L. donovani*-infected BALB/c mice was able to downregulate the expression of both *IL-1β* and *IL-1rn*, but not *IL-1α*, which was found to be upregulated (Fig. 7).

Mice treated with 45 mg/kg b.w. of Ole (G1) exhibited a significant 20.4-fold (*P* = 0.025) decrease in *IL-1β* gene expression compared to the infected control group (G5), while the HePC treatment did not significantly alternate the *IL-1β* expression (Fig. 7). Moreover, mice treated with 15 and 5 mg/kg b.w. of Ole (G2 and G3) showed a tendency of lower *IL-1β* expression, 7.69-fold (*P* = 0.1) and 20-fold (*P* = 0.055), respectively, in comparison to the infected control. Clearly the *IL-1β* gene expression was similar among the infected (G5) and healthy control (G6) groups.

Moreover, in *L. donovani*-infected BALB/c mice (G5), *IL-1rn* gene expression was 1.7 times diminished (*P* ≤ 0.1) compared to the healthy control group (G6). However, Ole-treated mice exhibited a tendency for an additional drastic IL-1rn downregulation. Ole treatments of 45, 15, and 5 mg/kg b.w. caused a 20-fold (*P* = 0.055), 6.67-fold (*P* = 0.055) and 9.09-fold (*P* = 0.055) decrease in *IL-1rn* expression, respectively, compared to the infected control group (G5; Fig. 7). HePC-treatment also caused suppressed *IL-1rn* expression compared to the infected control group (G5) exhibiting a 5.88-fold downregulation (*P* = 0.037).

The *IL-1α* gene expression was regulated similarly to *IL-1rn* due to *L. donovani* infection. Healthy BALB/c mice (G6) exhibited a 2-fold augmented *IL-1α* transcription compared to the infected control group (G5). On the other hand, Ole-treated mice exhibited significant increases in *IL-1α* expression. Ole-treated (45, 15 and 5 mg/kg b.w.) mice showed a 2.67-fold (*P* = 0.1), 6.22-fold (*P* = 0.004) and 4.18-fold (*P* = 0.037) increase, respectively, compared to the infected control group (G5) (Fig. 7). The finding that Ole induces the upregulation of *IL-1α* gene is important since treatment of *L. donovani*-infected mice with HePC (G4), showed reduced, even though not significantly *IL-1α* expression.

### Levels of *TNF-α*, *IFN-γ, TGF-β1, IL-12β* and *IL-10* gene expression

**Fig. 8:**
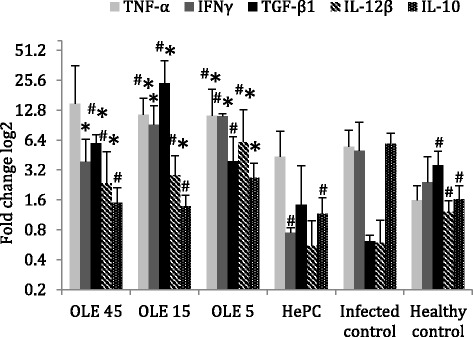
Fold changes log2 of the *TNF-α, IFN-γ*, *TGF-β1*, *IL-12β* and *IL-10* gene expression in splenocytes of *L. donovani*-infected BALB/c mice, 3 days after termination of treatments with Ole and HePC. ^#^ and ^*^ indicate significant differences compared to infected and positive (HePC-treated) control groups, respectively

T-cell immune responses are determined by the predominance of specific cytokines since they regulate T-cell differentiation [43]. It is known that *IL-12β* gene is responsible for the expression of IL-12p40, a basic subunit of IL-12 and IL-23 cytokines. IL-12 is necessary for Th1 polarization and IL-23 is necessary for leishmanicidal activity in chronic experimental leishmaniasis [44]. In our in vivo study, *L. donovani* infection (G5) drove into a tendency for repression of *IL-12β* expression that reached 51 %  (*P* = 0.055) in comparison to the healthy control group (G6; Fig. 8). On the contrary, two of the experimental groups that received Ole (G2 and G3) exhibited elevated *IL-12β* gene expression demonstrating a 4.77-fold (*P* = 0.025) and 10.15-fold (*P* = 0.025) statistically significant increase, respectively, compared to the infected control group (G5; Fig. 8). Ole-treated mice of group G1 (45 mg/kg b.w.) exhibited a 3.95-fold (*P* = 0.1) *IL-12β* upregulation in comparison to the infected control. However, HePC treatment was not able to promote the *IL-12β* expression over the infected control (G5).

Changes in *IL-10* gene expression in relation to IL-12 expression indicate the orientation of the polarized cell-mediated immunity towards a Th2 immune response. *Leishmania donovani*-infected mice (G5) exhibited a tendency for increased levels of *IL-10* gene transcription that reached 3.63-times higher levels than in the healthy control group (G6; *P* = 0.068; Fig. 8). This elevated expression of *IL-10* gene in splenocytes of infected mice was not present in treated mice. More specifically, mice treated with Ole (45, 15 and 5 mg/kg b.w.) showed a 3.85-fold (*P* = 0.1), 4.17-fold (*P* = 0.078) and 2.22-fold (*P* > 0.1) decrease, respectively, compared to the infected control group (G5; Fig. 8). Although mice treated with HePC did not overexpress *IL-12β*, they exhibited a 5.08-fold (*P* = 0.028) significant downregulation in *IL-10* transcription compared to the infected control group (G5).

The *IFN-γ* gene expression in mixed splenocytes of *L. donovani*-infected BALB/c mice followed the pattern of *IL-12β* expression (Fig. 8). *L.donovani*-infected mice (G5) showed a 2-fold upregulation of *IFN-γ* in comparison to the healthy control group (G6). Among Ole-treated mice, only mice treated with 5 mg/kg b.w. of Ole exhibited a significant positive fold change (2.25-fold, *P* = 0.025) in *IFN-γ* gene expression compared to the infected control group (G5), whereas HePC treatment downregulated significantly the *IFN-γ* expression (6.64-fold change; *P* = 0.004).

As shown previously, NO production can also be modulated by a IFN-γ-independent and TNF-α-dependent mechanism, which is orchestrated by IL-12 [6]. In this study, we also found that *TNF-α* expression was upregulated in mice treated with 45 mg/kg b.w. of Ole, which showed a 2.73-fold change (*P *= 0.037) compared to the infected control group (G5; Fig. 8). Mice treated with 15 and 5 mg/kg b.w. of Ole tended into a 2.1-fold (*P* = 0.078) and a 2.06-fold (*P* = 0.1) increase of *TNF-α* expression in comparison to the infected control mice. Moreover, *L. donovani*-infected mice also exhibited a 3.43-fold over transcription of *TNF-α* expression compared to the healthy control group (G6), since TNF-α also participates in the inflammatory process. HePC treatment had no effect in *TNF-α* expression compared to the infected control group (G5).

Finally, the expression of *TGF-β1* gene tended into repression (5.18-fold; *P* = 0.055) due to *L. donovani* infection (Fig. 8). On the other hand, Ole-treated groups (15 and 5 mg/kg b.w.) demonstrated a 38.63-fold (*P* = 0.01) and 6,34-fold (*P* = 0.037) upregulated transcription of *TGF-β1* in comparison to the infected control group (G5). Mice treated with 45 mg/kg b.w. presented a tendency of 9.66-fold (*P* = 0.1) increased *TGF-β1* gene transcription levels. Finally, HePC treatment did not significantly alter the *TGF-β1* expression over *L. donovani* infection.

### *Tbx21* and *GATA3* transcription factors gene expression

The modulating action of Ole in the expression of specific genes in splenocytes of *L. donovani*-infected BALB/c mice*,* prompted us to investigate the expression of transcription factors, Tbx21 and GATA3, which are responsible for cell-mediated immune response polarization toward the Th1 or Th2 phenotype, respectively [4].

**Fig. 9:**
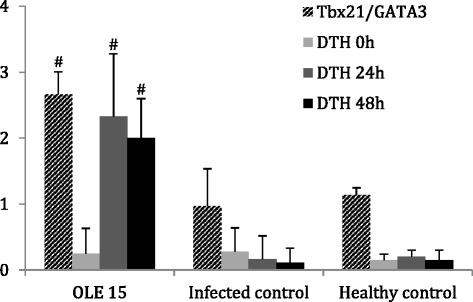
*Tbx21*/*GATA3* gene expression ratios in splenocytes (cross-hatched bars) and delayed type hypersensitivity (DTH, shaded bars) in *L. donovani*-infected BALB/c mice, untreated or treated with 15 mg/kg b.w. of Ole, measured at 3 days after treatment termination. The Y-axis represents both the Tbx21/GATA3 ratio (unitless) and the difference in skin thickness after the reaction (mm). At each time point in the DTH assay, the values represent the thickness after a reaction to the *Leishmania* antigen minus the thickness in the saline control. ^#^indicates significant differences compared to the infected control group

Three days after treatment termination the *Tbx21*/*GATA3* gene expression ratio was significantly increased in Ole-treated mice (Ole 15 mg/kg b.w.; 2.75-fold change; *P* = 0.004) compared to the infected control group (Fig. 9). This augmented *Tbx21*/*GATA3* ratio in Ole-treated mice was a result of downregulation in *GATA3* expression rather than the corresponding upregulation in *Tbx21* expression. It is noteworthy, that mice of the infected control group as well as of the healthy control group exhibited a similar *Tbx21*/*GATA3* ratio.

### Delayed type hypersensitivity (intradermal reaction to *L. donovani* promastigote lysate)

A DTH assay can detect the in vivo development of a specific cellular response against the intradermally inoculated antigen where a positive reaction is manifested by swelling and erythema at the site of administration. Three days after termination of treatment with 15 mg/kg of b.w. of Ole, we intradermally administered soluble *Leishmania* antigen in the footpad of *L. donovani*-infected as well as healthy control BALB/c mice (Fig. 9). After 24 h, the footpad swelling was 2.33 ± 0.95 mm in Ole-treated mice and 0.17 ± 0.35 mm in the infected control group (*P* = 0.001, Fig. 9). At 48 h, the differences between groups were sustained and this disclosed the in vivo operation of a parasite specific cell-mediated immunity (2 ± 0.60 mm in Ole-treated mice and 0.11 ± 0.22 mm in infected control mice; *P* < 0.0001).

## Discussion

Ole had been previously shown to exhibit antiparasitic activity against *L. donovani* amastigotes in J774A.1 macrophages. This finding is further associated with elevated Ole-induced intracellular and extracellular oxidative stress (ROS and NO production) in non-infected or infected J774A.1 macrophages, as shown in the present study. It is of great interest that Ole *per se* as well as its co-administration with LPS, generated limited oxidative stress in non-infected J774A.1 cells and these findings could probably be attributed to the different concentrations of Ole and to its *in vitro* antioxidant effect [45, 46]. In contrast, when J774A.1 cells were infected with *L. donovani* parasites, Ole induced significant production of ROS, although it has been reported that monocytes of patients with active visceral leishmaniasis exhibited decreased NADH-oxidase and NADPH-oxidase activity compared to healthy controls from endemic and non-endemic areas [16]. Data described in the present study showed that Ole at low concentrations behaved as an antioxidant, by reducing the production of ROS caused by the presence of LPS, whereas Ole at high concentrations, promoted J774A.1 activation by elevating the generalized oxidative stress (increased levels of ROS and NO production).

These results are in accordance with other studies of anti-leishmanial agents from natural resources. For example, *Piper betle* L. (*Piperaceae*) crude extract augmented ROS levels when incubated with *L. donovani*-infected J774A.1 cells following a time dependent increase up to 24 h [47]. However, this result conflicted with other studies, which highlighted the antioxidant activity of *P. betle* [48]. Moreover, luteolin, a well-studied plant derived biophenol, although showing a satisfactory action against *L. donovani* amastigotes [49], reduced NO production in RAW264.7 macrophages incubated with LPS [50]. Ole as well as resveratrol, are natural compounds with opposed actions, antioxidants or pro-oxidants. This dual action could possibly be attributed to their metabolic activation through a metal ion (e.g. Cu^+2^) chelation [51–54]. Indeed metal ions are typically present in biological systems or as contaminants in biological reagents and the chelation of these ions can produce toxic pro-oxidants, capable of inducing oxidative stress in infected macrophages.

Thus, the ability of several active compounds obtained from plant extracts or isolated compounds to act as growth inhibitors of promastigotes and amastigotes in in vitro systems, led to the investigation of their role in in vivo protocols that delineate their biological effect in depth. Numerous studies are focused on the mechanisms possessed by natural products used in traditional medicine, in order to affect the immune system. Their effect vary in inducing different immune mediators such as cytokines and chemokines resulting in the establishment of a protective immune response that will provide effective parasite elimination without producing excessive tissue destruction. Thereafter, we evaluated the effect of Ole in an in vivo murine model of visceral leishmaniasis. Our data showed that soon after Ole treatment termination (at 3 days), splenocytes obtained from *L. donovani*-infected BALB/c mice, exhibited significant intracellular ROS production and increased levels of NO in the supernatants of splenocytes and hepatocytes. Similarly, other natural products, such as asiaticoside and fucoidan, also produce high levels of ROS and NO in splenocytes of *L. donovani*-infected mice, and these findings explain the diminution of the parasite burden in the spleen [55, 56]. It is noteworthy that Ole induced more potent production of oxygen intermediates in *L. donovani*-infected mice, compared to HePC, the first oral drug approved for the treatment of visceral leishmaniasis. HePC was found to support solely the production of NO from spleen and liver cells at 3 days after the termination of treatment.

Excessive production of ROS may lead to oxidative stress, loss of cell function and ultimately apoptosis or necrosis [21]. Thus, a balance between oxidant and antioxidant intracellular systems is vital for cell function and regulation. Mammalian cells and *Leishmania* parasites are able to counteract oxidative stress through GSH and TSH production, respectively [57]. In our in vivo model of experimental visceral leishmaniasis, we found no significant changes in total or free GSH levels in the liver among all experimental groups (data not shown). This finding highlighted the notion that infected liver cells were protected from the oxidative stress induced by Ole. Previous studies have shown that Ole could in vitro replenish the GSH pool of J774A.1 macrophages by restoring glutathione reductase and peroxidase activity, as well as by inducing their mRNA expression [32]. In the present study, we examined the effect of Ole on the expression of the catalytic subunit of the glutamate-cysteine ligase (GCLC) in host splenocytes and parasite cells. GCLC is a rate-limiting enzyme in GSH and TSH synthesis in mammalian cells and parasites, respectively. Transgenic promastigotes heterozygous for *GCLC* (*L. donovani GCLC*^+/-^) exhibited diminished TSH levels, which rendered parasites more vulnerable to oxidative stress in vitro with decreased survival ability inside activated macrophages [23]. Moreover, other studies have shown differences in host and parasite *GCLC* gene regulation when sodium stibogluconate (SSG)-sensitive or SSG-resistant *L. donovani* parasites were treated with SSG [38]. The transcription regulation of *GCLC* gene is also found to be organ-specific since augmentation of murine *GCLC* expression only occurs in the spleen [38]. However, increases in m*GCLC* expression appeared to protect SSG-resistant *L. donovani* parasites, which abrogated the need for parasites to recruit additional “defensive measurements” such as Ld*GCLC* upregulation [38]. In the absence of SSG, SSG-resistant *L. donovani* parasites could downregulate m*GCLC* and upregulate the Ld*GCLC* expression. This capacity allowed parasites to elevate their oxidative stress resistance, because TSH has 600-fold greater affinity for NO^•^ compared to GSH [22, 38]. In our study, Ole-treated groups exhibited differential m*GCLC* and Ld*GCLC* expression, 3 days after treatment termination where Ld*GCLC* and m*GCLC* expression was downregulated and upregulated, respectively. On the contrary, in HePC-treated mice, as well as in infected control mice, m*GCLC* and Ld*GCLC* genes were regulated inversely. This expression profile supported the parasite need to dodge the oxidative burst with defensive mechanisms. In contrast, Ole appeared to function in both systems, because it maintained Ld*GCLC* expression at low levels, rendering parasites susceptible to oxidative burst, and at the same time, assisting the host cell in neutralizing ROS and RNI production.

NF-kB proteins are a family of transcription factors with great importance in inflammation and immunity [58]. ROS have been reported to both activate and to repress NF-kB signaling. At the cellular level, the *NF-kB2* gene, that encodes the NF-kB p100 subunit, is primarily upregulated by inflammatory lymphokines, such as IL-1β and TNF-α; however, the activation of NF-kB p50/p65 heterodimers by various stimuli (including ROS) can also autoregulate *NF-kB2* expression [59, 60]. In our study, *NF-kB2* expression in splenocytes from treated mice (Ole- or HePC-treated) remained at similar levels compared to the healthy control group, even though Ole-treated mice exhibited augmented oxidative stress, 3 days after treatment termination. In contrast, splenocytes from infected control mice exhibited upregulated expression of *NF-kB2* possibly due to the significant upregulation of *IL-1β* expression. Thus, all of the above results indicate that splenocytes of Ole-treated mice are adequate to eliminate intracellular parasites via ROS and NO-dependent mechanisms, in the absence of excessive inflammation in the environment of splenocytes, as indicated by the absence of *NF-kB2* upregulation and the enhanced *IL-1β* downregulation [21, 59].

Moreover, Ole treatment resulted in the downregulation of both *IL-1β* and *IL-1rn*, in combination with *IL-1α* upregulation. The above data indicate that in Ole-treated mice the only member of the IL-1 cytokine family available for binding to the IL-1 receptor was IL-1α, because IL-1β and IL-1rn were selectively downregulated, a finding that has not been described before in experimental visceral leishmaniasis [61]. In contrast, it is already known that IL-1α requires TNF-α for optimal induction of the Th1-type cytokine IL-12 in susceptible BALB/c mice [62], an effect that was also found in our study since Ole-treated mice overexpressed all three cytokines in their spleen. On the other hand, *L. donovani* parasites could not modulate IL-1α, in either susceptible BALB/c mice or resistant C3H/HeN mice [63]. Moreover, IL-1α seemed to be less important than IL-1β as an inflammatory cytokine in parasitic dissemination. Indeed, IL-1α^-/-^ mice were more resistant than IL-1β^-/-^ mice in the experimental visceral leishmaniasis model [34]. In addition, splenocytes from IL-1α^-/-^ mice failed to mount a Th1 polarization in the later stages of acute infection compared to IL-1β^-/-^ splenocytes [34]. The observed anti-inflammatory activity of Ole through repression of *IL-1β* gene seems to be very important in the first steps of experimental visceral leishmaniasis, since IL-1β was found to be in greater necessity for CD4^+^ polarization to the Th2 phenotype than IL-1α [34]. The Th2 cell-mediated immune response to *L. donovani* infection is triggered and established by IL-1β through a well-established mechanism that enables PGE2 induction via *COX-2* upregulation, [11, 64]. In this study, Ole suppressed the expression of *IL-1β*, an effect also found in LPS-stimulated human peripheral blood mononuclear cells, where Ole was incapable of altering IL-6, TNF-α and PGE2 levels [33].

In the present study, we demonstrated the ability of Ole to initiate a Th1 cell-mediated immune response in a murine model of visceral leishmaniasis. This immunomodulatory effect of Ole is highlighted in the IL-12/IL-10 ratio, which represents a marker of disease severity in leishamaniasis [65]. More specifically, all of the Ole-treated experimental groups exhibited significantly higher *IL-12β* and lower *IL-10* gene expression than the infected control group. The ability of splenocytes from Ole-treated BALB/c mice to express *IL-12β* is of paramount importance, as it has been previously shown that IL-12 is intrinsically linked to protection against visceral leishmaniasis [66]. Moreover, at this time point, we did not find an elevated *IL-12β* expression in HePC-treated mice. Furthermore, we found that Ole-treated mice exhibited elevated *TNF-α* and *IFN-γ* gene expression. Therefore, these elevated gene expression levels might be responsible for the diminished parasite burden, the augmented ROS and NO levels and the upregulated *iNOS* gene expression (data not shown) found in Ole-treated splenocytes.

On the other hand, we observed augmented expression of the *TGF-β1* gene which transcripts a cytokine characterized by its immunosuppressive activity [67, 68]. However, TGF-β was found to play a leading role in IL-12^-/-^ and IL-12^-/-^/IFN-γ^-/-^ C57BL/6 mice that could not mount a Th1 immune response [69]. Also, TGF-β-producing Tregs were essential for an effective secondary immune response against *Leishmania* parasites since Treg elimination in BALB/c mice resulted in a Th2 immunological phenotype and the deterioration of leishmaniasis [70].

All of the above findings indicate that Ole treatment in *L. donovani*-infected BALB/c mice could indirectly or directly modulate the immune response. This result is further confirmed by the highly elevated *Leishmania*-specific IgG2a/IgG1 ratios found in the serum of Ole-treated mice. This IgG2a overproduction was also evident at 6 weeks after treatment termination. In contrast, infected control mice had IgG2a/IgG1 ratios close to 0.5, which indicate a dominance of the Th2 immune response. This immunoglobin production switch from IgG1 to IgG2a is typically driven by IL-12 and IFN-γ produced by Th1 cells [71–73].

We further assessed the Th1/Th2 mixed cellular immune response by determining the gene expression of the Tbx21 and GATA3 transcription factors. An experimental model of visceral leishmaniasis in BALB/c mice typically exhibits a Th1/Th2 mixed immunological response. The *Tbx21*/*GATA3* gene expression ratio reflects the prevailing polarization. *GATA3* gene expression is predominantly important for Th2 immune response, because its inhibition will result in *Tbx21* expression, which is stimulated by Th1 cytokines, like IL-12 and IFN-γ [5, 6]. In our in vivo experiments, Ole treatment caused an upregulation of *Tbx21* over *GATA3*, which enables a dominance of Th1 cellular immune response in the spleen. This finding was consistent with the cytokine gene expression profile described previously. Next, we performed the DTH test, as an index of cell-mediated immunity, in mice treated with Ole, to assess its effect in promoting enhanced phagocytosis of the infected host leading to effective parasite elimination. Strong DTH responses significantly reduce parasite burden and thus are important in host defense mechanisms against *Leishmania* infections. We found that BABL/c mice treated with Ole developed positive DTH response maximized at 24 h that is predictive of their capacity to resolve the infection.

## Conclusions

In conclusion, we showed that Ole is able to induce ROS production in an in vitro, as well as in an in vivo model of visceral leishmaniasis. Moreover, Ole augments NO production in *ex vivo* cultures of spleen and liver cells. The induced oxidative burst is mediated in the host by the regulation of glutathione-related genes, and at the same time, Ole renders parasites vulnerable by diminishing the expression of their respective TSH-producing enzymes. These diverse activities are accompanied by the intense downregulation of *IL-1β* and *IL-1rn* genes, but not *IL-1α*, which allows the *NF-kB2* expression despite the oxidative stress. Ole treatment downregulates *IL-10* gene expression, and in combination with *IL-12β* overexpression, drives a predominant Th1 polarization that is accompanied with *IFN-γ* and *TNF-α* overtranscription. This *Leishmania* protective cell-mediated immune response was clearly demonstrated by the positive DTH reaction, the ratio of *Tbx21*/*GATA3* transcription factors and *Leishmania*-specific IgG2a/IgG1 antibodies, indicating the operation of a Th1 type of immune response. On the other hand HePC was unable to modulate the majority of the genes tested since, as mentioned previously, its main route of action is through lipid metabolism [74]. The immunomodulatory effect of HePC seems to involve IFN-γ receptor and IFN-γ responsiveness through STAT1 phosphorylation taking advantage of the endogenous IFN-γ levels [75]. Thus, the diminished parasite burdens that both Ole and HePC presented in our previous study [29], is a result of different mechanisms. Finally, the present study illustrated the promising therapeutic properties of Ole as a natural product with leishmanicidal activity and immunomodulatory effects that are mainly triggered by its anti-inflammatory and antioxidative effects towards the benefit of the infected host.

## Abbreviations

CM-H_2_DCFDA, a chloromethyl derivative of 2′, 7′-dichlorodihydrofluorescein diacetate; COX-2, cyclooxygenase-2; DTH, delayed-type hypersensitivity response; ELISA, enzyme-linked immunosorbent assay; FACS, fluorescence-activated cell sorting; FBS, fetal bovine serum; GAPDH, glyceraldehyde dehydrogenase of the 3-phosphatase; GATA3, trans-acting T-cell-specific transcription factor; GCLC, calalytic subunit of glutamate-cysteine ligase; gMFI, geometric mean fluorescence intensity; GSH, glutathione; H_2_O_2_, hydrogen superoxide; HePC, hexadecylphosphocholine or miltefosine; HEPES, 4-(2-hydroxyethyl)-1-piperazineethanesulfonic acid; HPI, Hellenic Pasteur Institute; IC_50_, half maximal inhibitory concentration; IFN-γ, interferon-γ; IL-10, interleukin-10; IL-12β, subunit 2 of interleukin-12; IL-1rn, interleukin-1 receptor antagonist; IL-1α, interleukin-1α; IL-1β, interleukin-1β; iNOS, inducable nitric oxide synthase; LdAtub, *L. donovani* a-tubulin; LPS, lipopolysaccharide; MAPK, mitogen-activated protein kinases; NADH, Nicotinamide adenine dinucleotide; NADPH, Nicotinamide adenine dinucleotide phosphate; NF-kB2, subunit 2 of nuclear factor kappa-B; NO, nitric oxide; Ole, oleuropein; PBS, phosphate-buffered saline; PGE2, prostaglandin E2; RNI, reactive nitrogen intermediates; ROS, reactive oxygen species; SLA, soluble *Leishmania* antigen; STAT1, signal transducer and activator of transcription 1; Tbx21, T-box transcription factor; TGF-β1, transforming growth factor beta-1; Th, T helper; TNF-α, tumor necrosis factor-α; Tregs, regulatory T cells; TSH, trypanothione; USFDA, United States Food and Drug Administration Agency

## References

[1] Mukbel RM, Patten C, Gibson K, Ghosh M, Petersen C, Jones DE (2007). Macrophage killing of *Leishmania amazonensis* amastigotes requires both nitric oxide and superoxide. Am J Trop Med Hyg.

[2] Rodrigues IA, Mazotto AM, Cardoso V, Alves RL, Amaral AC, Silva JR (2015). Natural products: Insights into leishmaniasis inflammatory response. Mediators Inflamm.

[3] Piscopo TV, Mallia Azzopardi C (2007). Leishmaniasis. Postgrad Med J.

[4] Kanhere A, Hertweck A, Bhatia U, Gokmen MR, Perucha E, Jackson I (2012). T-bet and GATA3 orchestrate Th1 and Th2 differentiation through lineage-specific targeting of distal regulatory elements. Nat Commun.

[5] Chakir H, Wang H, Lefebvre DE, Webb J, Scott FW (2003). T-bet/GATA-3 ratio as a measure of the Th1/Th2 cytokine profile in mixed cell populations: predominant role of GATA-3. J Immunol Methods.

[6] Taylor AP, Murray HW (1997). Intracellular antimicrobial activity in the absence of interferon-gamma: effect of interleukin-12 in experimental visceral leishmaniasis in interferon-gamma gene-disrupted mice. J Exp Med.

[7] Wahl SM, Swisher J, McCartney-Francis N, Chen W (2004). TGF-beta: the perpetrator of immune suppression by regulatory T cells and suicidal T cells. J Leukoc Biol.

[8] Melby PC, Tabares A, Restrepo BI, Cardona AE, McGuff HS, Teale JM (2001). *Leishmania donovani*: evolution and architecture of the splenic cellular immune response related to control of infection. Exp Parasitol.

[9] Kaye PM, Aebischer T (2011). Visceral leishmaniasis: immunology and prospects for a vaccine. Clin Microbiol Infect.

[10] Gorak PM, Engwerda CR, Kaye PM (1998). Dendritic cells, but not macrophages, produce IL-12 immediately following *Leishmania donovani* infection. Eur J Immunol.

[11] Matte C, Maion G, Mourad W, Olivier M (2001). *Leishmania donovani*-induced macrophages cyclooxygenase-2 and prostaglandin E2 synthesis. Parasite Immunol.

[12] Kuroda E, Sugiura T, Zeki K, Yoshida Y, Yamashita U (2000). Sensitivity difference to the suppressive effect of prostaglandin E2 among mouse strains: a possible mechanism to polarize Th2 type response in BALB/c mice. J Immunol.

[13] Stafford JL, Neumann NF, Belosevic M (2002). Macrophage-mediated innate host defense against protozoan parasites. Crit Rev Microbiol.

[14] Channon JY, Roberts MB, Blackwell JM (1984). A study of the differential respiratory burst activity elicited by promastigotes and amastigotes of *Leishmania donovani* in murine resident peritoneal macrophages. Immunology.

[15] Lodge R, Diallo TO, Descoteaux A (2006). * Leishmania donovani* lipophosphoglycan blocks NADPH oxidase assembly at the phagosome membrane. Cell Microbiol.

[16] Kumar P, Pai K, Pandey HP, Sundar S (2002). NADH-oxidase, NADPH-oxidase and myeloperoxidase activity of visceral leishmaniasis patients. J Med Microbiol.

[17] Wilkins-Rodriguez AA, Escalona-Montano AR, Aguirre-Garcia M, Becker I, Gutierrez-Kobeh L (2010). Regulation of the expression of nitric oxide synthase by *Leishmania mexicana* amastigotes in murine dendritic cells. Exp Parasitol.

[18] MacMicking J, Xie QW, Nathan C (1997). Nitric oxide and macrophage function. Annu Rev Immunol.

[19] Krauth-Siegel RL, Comini MA (2008). Redox control in trypanosomatids, parasitic protozoa with trypanothione-based thiol metabolism. Biochim Biophys Acta.

[20] Monostori P, Wittmann G, Karg E, Turi S (2009). Determination of glutathione and glutathione disulfide in biological samples: an in-depth review. J Chromatogr B Analyt Technol Biomed Life Sci.

[21] Gloire G, Legrand-Poels S, Piette J (2006). NF-kappaB activation by reactive oxygen species: fifteen years later. Biochem Pharmacol.

[22] Bocedi A, Dawood KF, Fabrini R, Federici G, Gradoni L, Pedersen JZ (2010). Trypanothione efficiently intercepts nitric oxide as a harmless iron complex in trypanosomatid parasites. FASEB J.

[23] Mukherjee A, Roy G, Guimond C, Ouellette M (2009). The gamma-glutamylcysteine synthetase gene of Leishmania is essential and involved in response to oxidants. Mol Microbiol.

[24] Georgopoulou K, Smirlis D, Bisti S, Xingi E, Skaltsounis L, Soteriadou K (2007). In vitro activity of 10-deacetylbaccatin III against *Leishmania donovani* promastigotes and intracellular amastigotes. Planta Med.

[25] do Socorro SRMS, Mendonca-Filho RR, Bizzo HR, de Almeida Rodrigues I, Soares RM, Souto-Padron T (2003). Antileishmanial activity of a linalool-rich essential oil from *Croton cajucara*. Antimicrob Agents Chemother.

[26] Torres-Santos EC, Moreira DL, Kaplan MA, Meirelles MN, Rossi-Bergmann B (1999). Selective effect of 2′,6′-dihydroxy-4′-methoxychalcone isolated from *Piper aduncum* on *Leishmania amazonensis*. Antimicrob Agents Chemother.

[27] Patricio FJ, Costa GC, Pereira PV, Aragao-Filho WC, Sousa SM, Frazao JB (2008). Efficacy of the intralesional treatment with *Chenopodium ambrosioides* in the murine infection by *Leishmania amazonensi*s. J Ethnopharmacol.

[28] Bhattacharya S, Ghosh P, De T, Gomes A, Dungdung SR. In vivo and in vitro antileishmanial activity of *Bungarus caeruleus* snake venom through alteration of immunomodulatory activity. Exp Parasitol. 2013;135(1):126-33. 10.1016/j.exppara.2013.06.006 23830987

[29] Kyriazis JD, Aligiannis N, Polychronopoulos P, Skaltsounis AL, Dotsika E (2013). Leishmanicidal activity assessment of olive tree extracts. Phytomedicine.

[30] Han J, Talorete TP, Yamada P, Isoda H (2009). Anti-proliferative and apoptotic effects of oleuropein and hydroxytyrosol on human breast cancer MCF-7 cells. Cytotechnology.

[31] Cardeno A, Sanchez-Hidalgo M, Rosillo MA, Alarcon de la Lastra C (2013). Oleuropein, a secoiridoid derived from olive tree, inhibits the proliferation of human colorectal cancer cell through downregulation of HIF-1alpha. Nutr Cancer.

[32] Masella R, Vari R, D’Archivio M, Di Benedetto R, Matarrese P, Malorni W (2004). Extra virgin olive oil biophenols inhibit cell-mediated oxidation of LDL by increasing the mRNA transcription of glutathione-related enzymes. J Nutr.

[33] Miles EA, Zoubouli P, Calder PC (2005). Differential anti-inflammatory effects of phenolic compounds from extra virgin olive oil identified in human whole blood cultures. Nutrition.

[34] Voronov E, Dotan S, Gayvoronsky L, White RM, Cohen I, Krelin Y (2010). IL-1-induced inflammation promotes development of leishmaniasis in susceptible BALB/c mice. Int Immunol.

[35] Bhowmick S, Ali N (2009). Identification of novel *Leishmania donovani* antigens that help define correlates of vaccine-mediated protection in visceral leishmaniasis. PLoS One.

[36] Bankoti R, Stager S (2012). Differential regulation of the immune response in the spleen and liver of mice infected with *Leishmania donovani*. J Trop Med.

[37] Koutsoni O, Barhoumi M, Guizani I, Dotsika E (2014). Leishmania eukaryotic initiation factor (LeIF) inhibits parasite growth in murine macrophages. PLoS One.

[38] Carter KC, Hutchison S, Henriquez FL, Legare D, Ouellette M, Roberts CW (2006). Resistance of *Leishmania donovani* to sodium stibogluconate is related to the expression of host and parasite gamma-glutamylcysteine synthetase. Antimicrob Agents Chemother.

[39] Decuypere S, Rijal S, Yardley V, De Doncker S, Laurent T, Khanal B (2005). Gene expression analysis of the mechanism of natural Sb(V) resistance in *Leishmania donovani* isolates from Nepal. Antimicrob Agents Chemother.

[40] Livak KJ, Schmittgen TD (2001). Analysis of relative gene expression data using real-time quantitative PCR and the 2(-Delta Delta C(T)) method. Methods.

[41] Agallou M, Athanasiou E, Koutsoni O, Dotsika E, Karagouni E (2014). Experimental validation of multi-epitope peptides including promising MHC Class I- and II-restricted epitopes of four known *Leishmania infantum* proteins. Front Immunol.

[42] Omar SH (2010). Oleuropein in olive and its pharmacological effects. Sci Pharm.

[43] Sharma U, Singh S (2009). Immunobiology of leishmaniasis. Indian J Exp Biol.

[44] Murray HW, Tsai CW, Liu J, Ma X (2006). Responses to *Leishmania donovani* in mice deficient in interleukin-12 (IL-12), IL-12/IL-23, or IL-18. Infect Immun.

[45] Visioli F, Poli A, Gall C (2002). Antioxidant and other biological activities of phenols from olives and olive oil. Med Res Rev.

[46] de la Puerta R, Martinez Dominguez ME, Ruiz-Gutierrez V, Flavill JA, Hoult JR (2001). Effects of virgin olive oil phenolics on scavenging of reactive nitrogen species and upon nitrergic neurotransmission. Life Sci.

[47] Misra P, Kumar A, Khare P, Gupta S, Kumar N, Dube A (2009). Pro-apoptotic effect of the landrace Bangla Mahoba of Piper betle on *Leishmania donovani* may be due to the high content of eugenol. J Med Microbiol.

[48] Choudhary D, Kale RK (2002). Antioxidant and non-toxic properties of Piper betle leaf extract: in vitro and in vivo studies. Phytother Res.

[49] Mittra B, Saha A, Chowdhury AR, Pal C, Mandal S, Mukhopadhyay S (2000). Luteolin, an abundant dietary component is a potent anti-leishmanial agent that acts by inducing topoisomerase II-mediated kinetoplast DNA cleavage leading to apoptosis. Mol Med.

[50] Choi EY, Jin JY, Choi JI, Choi IS, Kim SJ (2011). Effects of luteolin on the release of nitric oxide and interleukin-6 by macrophages stimulated with lipopolysaccharide from *Prevotella intermedia*. J Periodontol.

[51] Cao G, Sofic E, Prior RL (1997). Antioxidant and prooxidant behavior of flavonoids: structure-activity relationships. Free Radic Biol Med.

[52] Halliwell B (2008). Are polyphenols antioxidants or pro-oxidants? What do we learn from cell culture and in vivo studies?. Arch Biochem Biophys.

[53] Andrikopoulos NK, Kaliora AC, Assimopoulou AN, Papageorgiou VP (2002). Inhibitory activity of minor polyphenolic and nonpolyphenolic constituents of olive oil against in vitro low-density lipoprotein oxidation. J Med Food.

[54] Ahmad A, Farhan Asad S, Singh S, Hadi SM (2000). DNA breakage by resveratrol and Cu(II): reaction mechanism and bacteriophage inactivation. Cancer Lett.

[55] Bhaumik SK, Paul J, Naskar K, Karmakar S, De T (2011). Asiaticoside induces tumour-necrosis-factor-alpha-mediated nitric oxide production to cure experimental visceral leishmaniasis caused by antimony-susceptible and -resistant *Leishmania donovani* strains. J Antimicrob Chemother.

[56] Kar S, Sharma G, Das PK (2011). Fucoidan cures infection with both antimony-susceptible and -resistant strains of *Leishmania donovani* through Th1 response and macrophage-derived oxidants. J Antimicrob Chemother.

[57] Turrens JF (2004). Oxidative stress and antioxidant defenses: a target for the treatment of diseases caused by parasitic protozoa. Mol Aspects Med.

[58] Hayden MS, Ghosh S (2008). Shared principles in NF-kappaB signaling. Cell.

[59] Morgan MJ, Liu ZG (2011). Crosstalk of reactive oxygen species and NF-kappaB signaling. Cell Res.

[60] de Wit H, Dokter WH, Koopmans SB, Lummen C, van der Leij M, Smit JW (1998). Regulation of p100 (NFKB2) expression in human monocytes in response to inflammatory mediators and lymphokines. Leukemia.

[61] Gazzinelli RT, Talvani A, Camargo MM, Santiago HC, Oliveira MA, Vieira LQ (1998). Induction of cell-mediated immunity during early stages of infection with intracellular protozoa. Braz J Med Biol Res.

[62] Shibuya K, Robinson D, Zonin F, Hartley SB, Macatonia SE, Somoza C (1998). IL-1 alpha and TNF-alpha are required for IL-12-induced development of Th1 cells producing high levels of IFN-gamma in BALB/c but not C57BL/6 mice. J Immunol.

[63] Delfino D, Chiofalo MS, Riggio G, Angelici MC, Gramiccia M, Gradoni L (1995). Induction of interleukin 1 alpha in murine macrophages infected in vitro with different species and strains of *Leishmania*. Microb Pathog.

[64] Gregory DJ, Sladek R, Olivier M, Matlashewski G (2008). Comparison of the effects of *Leishmania major* or *Leishmania donovani* infection on macrophage gene expression. Infect Immun.

[65] Mukherjee S, Mukherjee B, Mukhopadhyay R, Naskar K, Sundar S, Dujardin JC (2014). Imipramine exploits histone deacetylase 11 to increase the IL-12/IL-10 ratio in macrophages infected with antimony-resistant *Leishmania donovani* and clears organ parasites in experimental infection. J Immunol.

[66] Engwerda CR, Murphy ML, Cotterell SE, Smelt SC, Kaye PM (1998). Neutralization of IL-12 demonstrates the existence of discrete organ-specific phases in the control of *Leishmania donovani*. Eur J Immunol.

[67] Gorelik L, Constant S, Flavell RA (2002). Mechanism of transforming growth factor beta-induced inhibition of T helper type 1 differentiation. J Exp Med.

[68] Gorelik L, Fields PE, Flavell RA (2000). Cutting edge: TGF-beta inhibits Th type 2 development through inhibition of GATA-3 expression. J Immunol.

[69] Wilson ME, Recker TJ, Rodriguez NE, Young BM, Burnell KK, Streit JA (2002). The TGF-beta response to *Leishmania chagasi* in the absence of IL-12. Eur J Immunol.

[70] Aseffa A, Gumy A, Launois P, MacDonald HR, Louis JA, Tacchini-Cottier F (2002). The early IL-4 response to *Leishmania major* and the resulting Th2 cell maturation steering progressive disease in BALB/c mice are subject to the control of regulatory CD4 + CD25+ T cells. J Immunol.

[71] Finkelman FD, Holmes J, Katona IM, Urban JF, Beckmann MP, Park LS (1990). Lymphokine control of in vivo immunoglobulin isotype selection. Annu Rev Immunol.

[72] Bretscher PA, Wei G, Menon JN, Bielefeldt-Ohmann H (1992). Establishment of stable, cell-mediated immunity that makes “susceptible” mice resistant to *Leishmania major*. Science.

[73] Kolbe L, Heusser C, Kolsch E (1991). Antigen dose-dependent regulation of B epsilon-memory cell expression. Int Arch Allergy Appl Immunol.

[74] Dorlo TP, Balasegaram M, Beijnen JH, de Vries PJ (2012). Miltefosine: a review of its pharmacology and therapeutic efficacy in the treatment of leishmaniasis. J Antimicrob Chemother.

[75] Wadhone P, Maiti M, Agarwal R, Kamat V, Martin S, Saha B (2009). Miltefosine promotes IFN-gamma-dominated anti-leishmanial immune response. J Immunol.

